# Predictors of Perinatal Mortality Associated with Placenta Previa and Placental Abruption: An Experience from a Low Income Country

**DOI:** 10.1155/2014/307043

**Published:** 2014-06-04

**Authors:** Yifru Berhan

**Affiliations:** College of Medicine and Health Sciences, Hawassa University, P.O. Box 1560, Hawassa, Ethiopia

## Abstract

A retrospective cohort study design was used to assess predictors of perinatal mortality in women with placenta previa and abruption between January 2006 and December 2011. Four hundred thirty-two women (253 with placenta previa and 179 with placental abruption) were eligible for analysis. Binary logistic regression, Kaplan-Meier survival curve, and receiver operating characteristic (ROC) curve were used. On admission, 77% of the women were anaemic (<12 gm/dL) with mean haemoglobin level of 9.0 ± 3.0 gm/dL. The proportion of overall severe anaemia increased from about 28% on admission to 41% at discharge. There were 50% perinatal deaths (neonatal deaths of less than seven days of age and fetal deaths after 28 weeks of gestation). In the adjusted odds ratios, lengthy delay in accessing hospital care, prematurity, anaemia in the mothers, and male foetuses were independent predictors of perinatal mortality. The haemoglobin level at admission was more sensitive and more specific than prematurity in the prediction of perinatal mortality. The proportion of severe anaemia and perinatal mortality was probably one of the highest in the world.

## 1. Introduction

Placenta previa (placenta implanted over the internal cervical os) and placental abruption (premature separation of normally implanted placenta) are the major causes of antepartum haemorrhage in the third trimester of pregnancies and major contributors of obstetric haemorrhage in general [1]. Each of these conditions has a prevalence rate of 0.5% to 2% in most parts of the world [2–4]. Because of the changes in the lower uterine segment length and placental migration as the pregnancy advances, the prevalence of placenta previa has an inverse relation to the gestational age [5]. In other words, it is suggested that reporting of placenta previa in early gestation is likely to overestimate its actual prevalence at term.

Placenta previa and placental abruption have long been recognized as major obstetric complications that result in maternal and fetal mortality as well as morbidity. The effect of these two bloody obstetric complications on perinatal health is multifactorial: blood loss, premature delivery, intrauterine growth restriction, the risk of perinatal asphyxia, the risk of sepsis, and hyperbilirubinemia [2, 6–8]. A Danish national cohort study was associated with an increased risk of neonatal mortality, prematurity, low Apgar scores, low birthweight, and transfer to a neonatal intensive care unit [9]. Several other studies from developing countries have also shown that pregnant women complicated by placenta previa are likely to have babies delivered preterm, low birth weight, asphyxiated, and requiring intensive neonatal care, while stillbirth or neonatal mortality may also occur [7, 10, 11]. To be specific, the risk of perinatal mortality in women with placental previa is estimated to be 4% to 8% but, when accompanied by prematurity, the death rate may increase to 50% [12].

On the other hand, the perinatal mortality in placental abruption cases may be as high as 20% to 47% [3, 13]. Specific to developed countries, a recent review has shown that 10–20% of all perinatal deaths are caused by placental abruption [14]. More than a decade back, studies in developed countries concluded that placental abruption had a profound impact on stillbirth [15, 16]. However, a large scale review in the United States in 2001 reported that the high perinatal mortality with abruption was mainly due to its strong association with preterm delivery [17]. But with the advancement of obstetric service, the perinatal mortality has been reported as decreasing [18, 19].

To the best of the author's knowledge, there is no published study that assessed placenta previa and placental abruption associated perinatal mortality and morbidity in Sub-Saharan Africa and in Ethiopia in particular. Thus, this study shades light on the most common complications of placenta previa and placental abruption with emphasis on the primary outcome predictors, the magnitude of perinatal mortality, and associated factors. Secondly, it gives a ground evidence on how soon or late women with placenta previa and placental abruption were coming to the hospital; what interventions were undertaken; and what was the condition of the neonates and mothers at discharge. In short, the findings of this study are instrumental to identify barriers and delays at the community and hospital level. The objective of this study was to determine the predictors of perinatal mortality associated with placenta previa and placental abruption.

## 2. Methods

This retrospective cohort study included all women with placenta previa and placental abruption who were admitted and managed in Hawassa University Hospital between January 2006 and December 2012. Hawassa University Hospital, located in the capital city of Southern Nation Nationalities and People Regional State of Ethiopia, has a catchment population of more than 16 million. During this study period, 9619 women gave birth in this hospital. Out of these, 511 women with singleton pregnancies were diagnosed to have antepartum haemorrhage (APH) at admission. Multiple births were excluded taking into account that they can have many other complications that might add to the complications attributed to the placenta previa or placental abruption independently.

Further scrutiny of each of the 511 APH labelled patients' charts revealed that there was change of the admission diagnoses through progressive evaluation of the pregnant women and examination of the placenta after delivery. As a result, 21 cases were later on identified as abortion (gestational age < 28 weeks) [20]; 29 cases were due to heavy show; 6 cases were diagnosed to have local causes (cervical infection, polyps, leech infestation, and cervical carcinoma); and 23 cases were excluded because of incomplete documentation. A total of 432 cases were identified as eligible for analysis.

The data sources for this study were individual patient's chart, admission, and discharge record books. Data related to the patients' age, residence, distance travelled for hospital admission, duration of hospital stay, obstetric history, clinical condition, and laboratory results at admission and discharge were collected. When a pregnant woman presented with dark and nonclotting vaginal bleeding after 28 weeks of gestation, placental abruption was diagnosed. The finding of retroplacental clot/bloody jell or depression in the maternal placental surface after delivery was taken as confirmation for atypically presented placenta previa. Whereas ultrasound report of placenta praevia was taken as a final diagnosis. For the purpose of this study, the degree of maternal anaemia and the survival of babies in the perinatal period were taken as primary outcome indicators.

In this study, APH was defined as any vaginal bleeding due to either placenta previa or placental abruption after 28 weeks of gestation before the delivery of the baby. The total time taken to reach the study hospital after the onset of APH symptoms (painless, bright red, and clotting vaginal bleeding in placenta previa; unexplained lower abdominal pain with or without dark nonclotting vaginal bleeding in placental abruption; and decreased or absent fetal kick in both) was taken as the duration before arrival. In this study, lengthy delay is to mean a period that lasted more than 12 hours since the onset of APH symptoms and long distance travelled implies 50 km or more to reach to the hospital. Gestational ages were categorized as very preterm (28–33 completed weeks), preterm (34–36 completed weeks), and term (37+ weeks). Perinatal status defined the fetal or early neonatal survival (from 28 weeks of pregnancy age up to the first 7 days of newborn age) [21] or otherwise after the onset of vaginal bleeding due to placenta previa or placental abruption.

The mean minimum normal haemoglobin level during pregnancy at sea level is 11-12 gm/dL [22]. In this study, the degree of anaemia was categorized as severe, moderate, mild, and no anaemia when the haemoglobin level was <7 gm/dL, 7–9.9 gm/dL, 10–11.9 gm/dL, and 12+ gm/dL, respectively. In this study, perinatal mortality and perinatal death are used interchangeably.

Ethical approval of this study was obtained from Hawassa University College of Medicine and Health Sciences Institutional Review Board. Written informed consent from the women included in this study was not required as only already registered data were used. Moreover, confidentiality and anonymity are assured by analyzing and disseminating the data in aggregate.

Data analysis was done using SPSS version 16.0 and Microsoft Office Excel 2010 computer software. Whisker and box plot was used to show the relation of perinatal mortality due to placenta previa and placental abruption with delay in arrival, gestational age, haemoglobin level, and fetal birth weight. The binary logistic regression model was applied to assess the association of perinatal mortality as a dependent variable with some selected variables as predictors. Kaplan-Meier survival curve with log rank test and receiver operating characteristic (ROC) curve (nonparametric method) were used to determine the strength of perinatal mortality predictors. *P* value < 0.05 was taken as indicator of statistically significant association.

## 3. Results

### 3.1. General Characteristics

Out of 432 antepartum haemorrhage cases included in this study, placenta previa and placental abruption constituted 253 (58.6%) and 179 (41.4%), respectively. The incidence of placenta previa and placental abruption in the study hospital during the study period was 2.6 and 1.9 per 1000 pregnant women. Placenta previa by type was totalis, partialis, and marginalis/low-lying which accounted for 151 (59.4%), 54 (21.5%), and 48 (19.1%), respectively. The degree of abruption (separation) for 107 (59.8%) was up to 50% or less and for the other 72 (40.2%) cases was over 50%. Twelve cases were diagnosed before the onset of vaginal bleeding. Otherwise, 420 (97.2%) women presented to the hospital after the onset of vaginal bleeding. Of which, 93 (21.5%) were admitted in a state of hemorrhagic shock (with unrecordable blood pressure and severe blood loss anaemia).

Nearly three-fourths of the women were in the age range of 20–34 years and more than three-fifths came from rural areas (Table 1). One hundred forty-five (33.6%) women traveled more than 100 km to reach the study hospital; the mean distance was 96 ± 93 km. When the proportions of mothers' age, residence, and distance travelled were stratified by placenta previa and placental abruption, there was no much difference. Women with placental abruption came relatively earlier than women with placenta previa: >12 hours delay before arrival in placenta previa and placental abruption group was 157 (62.1%) and 81 (45.%), respectively.

Overall, 238 (55.1%) women accessed the study hospital more than 12 hours after onset of their bleeding. The median delay in accessing the study hospital was 16 (interquartile range: 7–48) hours. In more than half of the cases (51.2%), with almost equivalent proportion in both placenta previa and placental abruption, the gestational ages had reached term (37+ weeks). On arrival at the hospital, 142 (32.9%) were fetal demise, and 53 (12.3%) foetuses had abnormal fetal heart rate (persistent bradycardia, persistent tachycardia, or irregularly fluctuating from normal range to bradycardia or tachycardia).

### 3.2. Degree of Anaemia

On admission, 334 (77.3%) of the women were found to have anaemia and the mean haemoglobin level was 9.0 ± 3.0 gm/dL (Table 2). The distribution of severe, moderate, and mild anaemia was almost proportional. Severe and mild anaemia was detected in the majority of women with placenta previa and placental abruption, respectively. When these women were discharged from the hospital, the severity of anaemia was found even worsening. The proportion of overall severe anaemia increased from 27.8% on admission to 41.2% at discharge and moderate anaemia from 27.1% to 30.6%.

**Figure 1:**
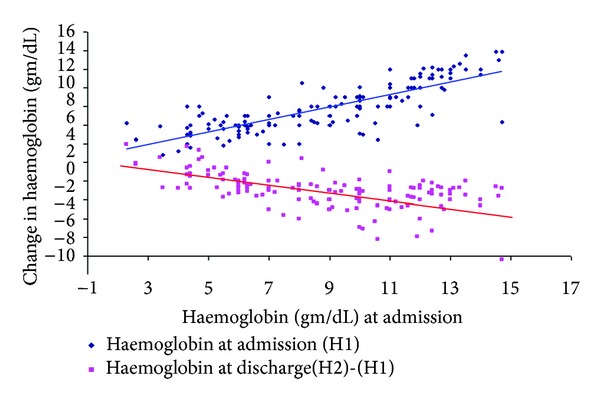
The change in haemoglobin level at the time of discharge from the level at the time of admission, 2006–2011, Hawassa University Hospital, Ethiopia.

In patients with placenta previa, severe anaemia increased from 33.2% on admission to 51.4% at discharge. In short, the proportion of anaemia increased from 201 (79.4%) to 234 (92.5%) in placenta previa cases and from 133 (74.3%) to 153 (85.5%) in placental abruption cases.  Figure 1 summarizes the change in haemoglobin level after delivery. In the majority of women, the haemoglobin levels at discharge were below the level on admission. Specifically, the drops in haemoglobin level were marked in those women who were better with the haemoglobin level >7gm/dL on admission.

### 3.3. Interventions in the Hospital

**Figure 2:**
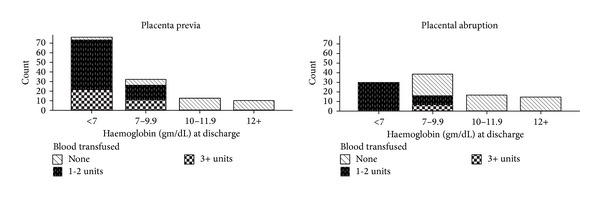
The distribution of anaemia* during the time of discharge from the hospital and the amount of blood transfused before achieving this amount of haemoglobin as paneled by type of antepartum haemorrhage (placenta previa and placental abruption). *Severe anaemia (haemoglobin < 7 gm/dL); moderate anaemia (Hb < 7–9.9 gm/dL); mild anaemia (Hb 10–11.9 gm/dL).

Table 3 shows the specific intervention carried out and fetal outcomes. It was possible to transfuse whole blood for 144 (33.3%) women. Of which, 106 (24.5%) were transfused with 1-2 units of blood (the transfusion criterion of the hospital is haemoglobin <7 gm/dL for blood loss anaemia with or without hemodynamic instability; one unit blood is equivalent to 450 mL). As presented in Figure 2, all women with placenta previa and the majority of women with placental abruption whose haemoglobin level <7 gm/dL at discharge were transfused 1-2 units of blood before having this amount of haemoglobin. Three women with placental abruption were not at all transfused despite having a haemoglobin level of <7 gm/dL.

Eighty-nine (20.6%) women were kept in the maternity ward before delivery for more than two days (median: 2.5 and IQR: 1–24 days). After delivery, 360 (83.3%) women were kept in the hospital for less than a week, with similar proportion in both placenta previa and placental abruption. Three-fourths of women gave birth by caesarean section. Active vaginal bleeding was the highest indication, 79.4% (258/325). One hundred forty-three (66.8%) of the perinatal deaths were delivered by caesarean section. Specifically, 92 (43%) caesarean sections were done for fetal demise and some other indications.

### 3.4. Perinatal Mortality

As shown in Table 3, there were 214 perinatal deaths, making the total perinatal mortality rate due to placenta previa and abruption about 495/1000 births or nearly 50%. The perinatal mortality rates associated with placenta previa and placental abruption were about 447/1000 births and 564/1000 births, respectively. Of the total perinatal deaths, 164 (38.0%) foetuses were found to be stillbirths (22 foetuses were admitted to the hospital with a positive fetal heart beat and died in utero) and 50 (11.6%) were early neonatal deaths. Out of the total perinatal deaths, placenta previa and placental abruption related deaths were 44.7% (113/253) and 56.4% (101/179), respectively. The proportion of stillbirths and early neonatal deaths in placenta previa and placental abruption were 34.4% versus 43.0% and 10.3% versus 13.4%, respectively.

In Table 4, the univariate analysis revealed that perinatal mortality due to placenta previa and placental abruption was strongly associated with long distance travel, lack of antenatal care, very low birth weight, and maternal severe anaemia. Other variables weakly associated with perinatal mortality due to placenta previa and placental abruption were maternal age above 20 years, rural residence, multiparity, and gestational age of 28–33 weeks during the time of delivery.

**Figure 3:**
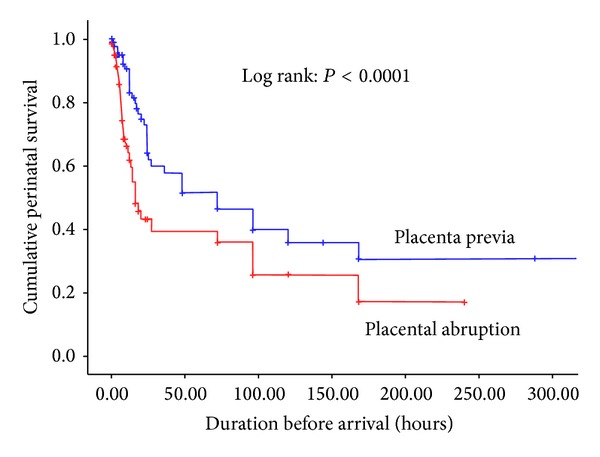
Kaplan-Meier estimates of the cumulative perinatal survival stratified by placenta previa and placental abruption, 2006–2011, Ethiopia.

For more than three-fifths of the total foetuses (62.5%), their birth weight was above 2500 gm and 259 (60.0%) of the foetuses were male, with comparable proportion in both placental abruption and placenta previa. The gestational age distribution of the perinatal deaths in weeks was 28–33 for 60 (28.0%), 34–36 for 52 (24.3%), and 37+ for 102 (47.7%). The Kaplan-Meier curve demonstrated that the perinatal mortality was the highest among placental abruption cases (log rank: *P* < 0.0001), and in both placenta previa and placental abruption cases, the survival curve dropped sharply in the early period, which may indicate that the majority of the babies died in the first 24 hours of onset of the bleeding (Figure 3).

**Figure 4:**
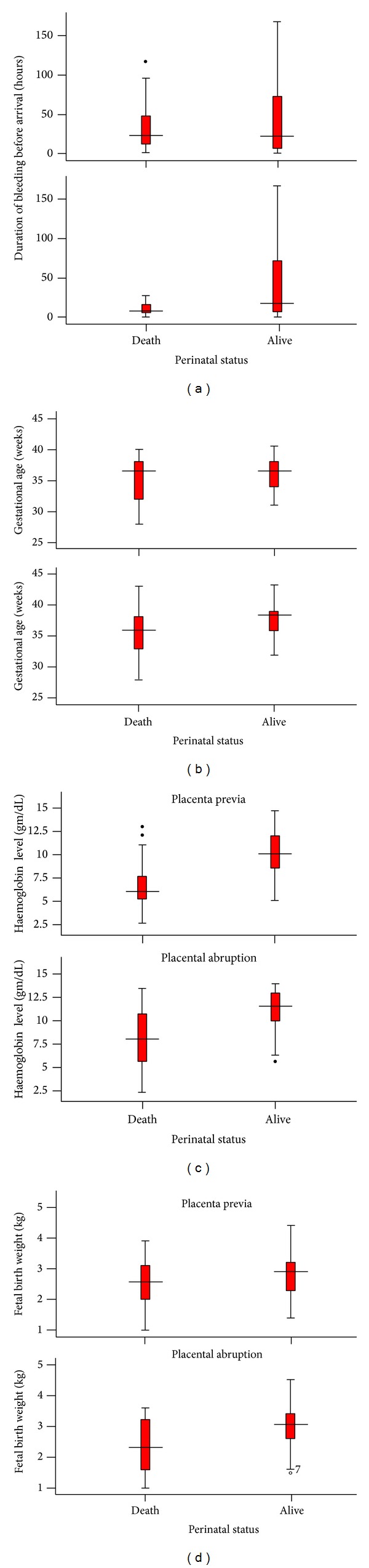
The relation of perinatal mortality with delay in arrival (a), gestational age (b), haemoglobin level (c), and fetal birth weight (d) as stratified by women with placenta previa and placental abruption, 2006–2011, Hawassa University Hospital, Ethiopia.

Figure 4 shows the relation of perinatal mortality with delay in arrival (a), gestational age (b), haemoglobin level (c), and fetal birth weight (d) as stratified by placenta previa and placental abruption. Both the median and IQR hours of delay among perinatal deaths were shorter than the survivors, which is another evidence to strengthen the findings in Figure 3. In women with placental abruption, the median gestational age and fetal birth weight were found to be lower in perinatal deaths than the survivors, whereas in women with placenta praevia, the median gestational ages of perinatal deaths and survivors were comparable. In both placental abruption and placenta previa, however, the median admission haemoglobin level of the cases resulting in perinatal deaths group was much lower than the survivors.

In Table 5, the crude odds ratios in the binary logistic regression have shown that long distance travel, lengthy delay in accessing the hospital, preterm gestational age, low blood pressure, anaemia on admission, and others were strongly associated with perinatal mortality. In the adjusted odds ratios, however, lengthy delay, preterm gestational age, anaemia level, being male, and delivered vaginally were independent predictors of perinatal mortality.

**Figure 5:**
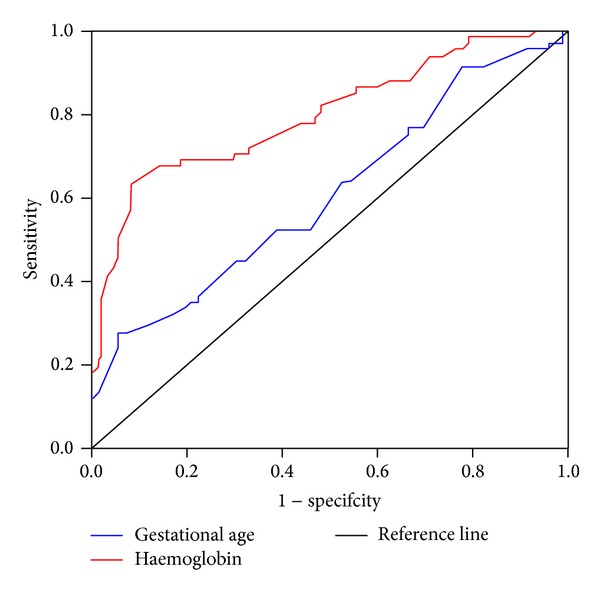
Receiver operating characteristics (ROC) curve comparing the sensitivity and specificity of haemoglobin level and gestational age on predicting perinatal mortality in women with placenta previa and placental abruption, 2006–2011, Hawassa University Hospital, Ethiopia.

Since the adjusted binary logistic regression model showed highly statistically significant association of perinatal mortality with low gestational age and haemoglobin level (OR 1.4 and 1.9, resp.), receiver operating characteristic curve (ROC) was depicted to test the two variables predictive validity in perinatal mortality (Figure 5). As the area under the curve shows, haemoglobin level on admission was more sensitive and more specific to determine perinatal mortality than the gestational age.

In this series of cases, there was no maternal death reported; couvelaire uterus was detected in 18 women with placental abruption who gave birth by caesarean section; and caesarean hysterectomy was done in 13 cases of placenta previa (12 for placental adherence and one for intractable postpartum haemorrhage).

## 4. Discussion

This study has shown that the perinatal mortality in women with placenta previa and placental abruption was about 50%, which is probably one of the highest in the world [3, 7, 10–13, 23]. Placenta previa associated perinatal mortality (about 45%) in particular was more than two- to threefold of some less developed countries [6, 23] and more than fifteenfold of a report from developed countries [9, 15, 24]. The perinatal deaths associated with placental abruption alone were also too high but were not as high as those in the recent report from Pakistan [25]. Similarly, the proportion of severely anaemic women both on admission and at discharge was surprisingly high, probably because of the big delay in reporting to the hospital, further blood loss after admission, and inadequate blood transfusion.

Unlike placental abruption, in which bleeding can be concealed and may be complicated with coagulopathy and further blood loss [26], bleeding due to placenta previa is immediately revealed and likely to make many of the pregnant women and their family seek prompt medical care. Relative ease of diagnosis in placenta previa is also an advantage for health professionals to undertake timely appropriate interventions and prevent further blood loss and associated maternal and perinatal complications. In this study, however, among women with placenta previa, more than one-third on admission and more than half at discharge were severely anaemic, which is probably the major reason why perinatal deaths were nearly proportional or higher than previously reported [3, 12, 13, 17–19, 23].

In general, the number of women with severe anaemia was quite large in both placenta previa and placental abruption group, which both contributed to about 50% of perinatal mortality. Among others, the adjusted binary logistic regression model also demonstrated that severe anaemia and delay in arrival were strong predictors for high perinatal mortality. Due to the excessive blood loss characteristic of these two obstetric problems, some degree of anaemia is inevitable regardless of the quality of care available in the country where the pregnant women reside.

Maternal anaemia resulting from bleeding was not reported to be contributors to perinatal mortality in some of the previous studies of placental previa and placental abruption [3, 12, 13, 23, 25]. This is probably because of the settings of advanced obstetric care and easy access of health facilities for the majority of pregnant women reported [9]; it might have resulted in a reduction of placenta previa and placental abruption related complications.

In the author's opinion, delay in arrival was probably at the epicenter of the whole problem of the patients included in this study. This is because, the longer pregnant women stay bleeding at home, the more they are likely to be severely anaemic. As a result, there is a high chance the foetuses will get asphyxiated or dead. Furthermore, since the hospital has been functioning as a referral with blood transfusion and operation facility, it is likely that it has more complicated placenta previa and placental abruption cases that had travelled long distance. Lengthier delay in arriving to the hospital was also observed among women with placenta previa than women with placental abruption, which was probably why many women with placenta previa were found in a severely anaemic state that has contributed to their significant overall perinatal deaths.

One may pose a question, why was there such significant delay in arrival and long distance travel? Since this study was based on registered data, it was not possible to get an exact answer for this question. However, from day to day observation in the hospital, it is possible to speculate that lack of health care seeking behavior (probably because of unawareness of available health services for this kind of problem), lack of local midwives for early referral, inability to get access to health facilities (usually due to financial problem and lack of ambulance service in the rural area), and delay in getting proper and satisfying treatment in health facilities including the hospital where these cases were managed might have contributed to high perinatal mortality and high blood loss anaemia. Whatever the barrier for early arrival was, the lengthy delay was the most likely factor for severe blood loss and high perinatal death.

Regardless of the delay in time, however, several studies have shown that placenta previa and placental abruption are known to triple the rate of perinatal mortality primarily due to prematurity [7, 8, 10, 12, 13, 17, 25]. In this study, however, as ROC curve showed, prematurity was not as sensitive and specific as haemoglobin level was in predicting perinatal mortality. The gestational age for more than two-thirds of the perinatal deaths was 34 weeks and above. But this finding has to be interpreted very cautiously. The relatively low perinatal deaths among very premature foetuses (28–33 weeks) may not be a true reflection as there were only a very small number of babies delivered prematurely. Taking into consideration the available intensive neonatal care and the reported neonatal survival rate, by definition, the viable gestational age for foetuses delivered in developed and developing countries including this study starts from 20–24 weeks [9, 15, 24] and 28 weeks [21], respectively. This could mean that if this study had included babies born before 28-week gestation with similar problems, prematurity might have been as well the leading associated problem for high perinatal mortality.

Otherwise, since the area under the curve is said to be an effective and combined measure of sensitivity and specificity for assessing the inherent validity of a predictor [27], above all, haemoglobin level on admission was the strongest predictor of perinatal mortality. The finding of about one-third of the women with severe anaemia and more than one-fifth presenting in shock on admission may also explain how critically ill the women themselves were. Probably it was because of such life threatening anaemia due to blood loss why nearly 80% of the women were subjected to give birth within 24 hours of their arrival to the hospital. Additionally, the finding of the majority of the women with further worsening anaemia indicate the continuous blood loss even after arrival to the hospital (probably due to the high caesarean delivery rate) and lack of adequate blood transfusion.

The explanation for the worsening haemoglobin level at discharge probably goes beyond the continuous blood loss even after arrival. The finding of more than half of women with placenta previa and more than one-fourth of women with placental abruption having severe anaemia at the time of discharge is an objective evidence to show the inadequate blood transfusion in the study hospital. Had it been possible, all women with severe anaemia should have been transfused with blood or had iron infusion and been discharged with haemoglobin level of 7 gm/dL and above. In a clinical setting such as the one in this hospital, like many others in low income countries, getting blood donor is a daily challenge and iron infusion is not yet in routine practice. As a result, many of severely anaemic patients are discharged after assessing only the overall clinical condition.

Limitations of this study are as follows: firstly the findings of this study could not be taken as incidence representing the general population in the hospital's catchment area. This is because the included mothers came mainly from a rural population where about 94% of pregnant women give birth at home [16]. Therefore, the reported cases in this study, if not representative, may reflect the magnitude of similar problems probably with worse outcomes back in the villages. Secondly, some asymptomatic or mild form of abruption may be under reported. Thirdly, some of the women might have variable degrees of anaemia in their pregnancy even before the onset of bleeding due to either placenta previa or placental abruption, which this study was not able to address. In Africa, severely anaemic pregnant women were reported to be between 1% and 5% [28] and 3% in Ethiopia [20]. Fourthly, some of the women discharged with severe anaemia might have had further complications which this study was not able to determine. Fifthly, although perinatal mortality is extended up to 28 weeks of postnatal period; this study was limited to the time the neonates were discharged from the hospital.

## 5. Conclusions

Low maternal haemoglobin level and delay in arrival were strong predictors of perinatal mortality due to placenta previa and placental abruption. The perinatal mortality was nearly 50% and 76.6% of these were stillbirths. The proportion of severe anaemia and perinatal mortality was probably one of the highest in the world. To reduce the burden of placenta previa and placental abruption related perinatal mortality, (1) comprehensive obstetric care including blood transfusion and iron infusion set up should be availed as close as possible to the villages where the study participants came from, and (2) there should be a strategy to let the community know the importance of antenatal care, maternal, and fetal risks of vaginal bleeding during pregnancy. Future studies should give emphasis on assessing the reasons for the delay in accessing and availing comprehensive obstetric care.

## Figures and Tables

**Table 1:** General characteristics of cases stratified by placenta previa and placental abruption, 2006–2011, Hawassa University Hospital, Ethiopia.

Variable	Placenta previa (*N* = 253)	Abruptio placenta (*N* = 179)	Total (*N* = 432)
Mothers' age in years			
<20	12 (4.7)	12 (6.7)	24 (5.6)
20–34	181 (71.6)	140 (78.2)	321 (74.3)
35+	60 (23.7)	27 (15.1)	87 (20.1)
Mothers' residence			
Urban	86 (34.0)	77 (43.0)	163 (37.7)
Rural	167 (66.0)	102 (57.0)	269 (62.3)
Distance traveled in km			
<50	98 (38.7)	80 (44.7)	178 (41.2)
50–100	70 (27.7)	39 (21.8)	109 (25.2)
101–200	37 (14.6)	42 (23.5)	79 (18.3)
>200	48 (19.0)	18 (10.0)	66 (15.3)
Duration before arrival in hours			
≤12	96 (37.9)	98 (54.7)	194 (44.9)
>12	157 (62.1)	81 (45.3)	238 (55.1)
Parity			
0	39 (15.4)	48 (26.8)	87 (20.1)
I–IV	111 (43.9)	93 (52.0)	204 (47.2)
V+	103 (40.7)	38 (21.2	141 (32.6)
Gestational age in weeks			
28–33	54 (21.3)	30 (16.8)	84 (19.4)
34–36	74 (29.3)	53 (29.6)	127 (29.4)
37+	125 (49.4)	96 (53.6)	221 (51.2)
Antenatal care			
Yes	153 (60.5)	128 (71.5)	281 (65.0)
No	100 (39.5)	51 (28.5)	151 (35.0)
Diastolic BP in mmHg			
Unrecordable	13 (5.1)	3 (1.7)	16 (3.7)
<60	65 (25.7)	12 (6.7)	77 (17.8)
60+	175 (69.2)	164 (91.6)	339 (78.5)
Fetal heart beat			
Negative	71 (28.1)	71 (39.7)	142 (32.9)
Abnormal	32 (12.6)	21 (11.7)	53 (12.3)
Normal	150 (59.3)	87 (48.6)	237 (54.8)

BP: blood pressure.

**Table 2:** The proportion and degree of anaemia at the time of admission and discharge as stratified by placenta previa and placental abruption, 2006–2011, Hawassa University Hospital, Ethiopia.

Haemoglobin in gm/dL	Mean	Severe anaemia (<7)	Moderate anaemia (7–9.9)	Mild anaemia (10–11.9)	No anaemia (12+)
At admission					
Total	**9.0 ± 3.0**	**120 (27.8)**	**117 (27.1)**	**97 (22.4)**	**98 (22.7)**
PP	8.7 ± 3.0	84 (33.2)	75 (29.6)	42 (16.6)	52 (20.6)
PA	9.5 ± 3.0	36 (20.1)	42 (23.5)	55 (30.7)	46 (25.7)
At discharge					
Total	**7.9 ± 2.6**	**178 (41.2)**	**132 (30.6)**	**77 (17.8)**	**44 (10.2)**
PP	7.6 ± 2.6	130 (51.4)	62 (24.5)	42 (16.6)	19 (7.5)
PA	8.4 ± 2.5	48 (26.8)	70 (39.1)	35 (19.6)	26 (14.5)

PP: placenta previa (*N* = 253).

PA: placental abruption (*N* = 179).

**Table 3:** Specific interventions and fetal outcome stratified by placenta previa and placental abruption, 2006–2011, Hawassa University Hospital, Ethiopia.

Variable	Placenta previa (*N* = 253)	Abruptio placenta (*N* = 179)	Total (*N* = 432)
Whole blood transfused			
1-2 units	67 (26.5)	39 (21.8)	106 (24.5)
3+ units	32 (12.6)	6 (3.4)	38 (8.8)
Hospital arrival to delivery time (days)			
≤1	188 (74.3)	155 (86.6)	343 (79.4)
2–7	34 (13.4)	15 (8.4)	49 (11.3)
≥8	31 (13.3)	9 (5.0)	40 (9.3)
Hospital stay after delivery (days)			
≤6	211 (83.4)	149 (83.2)	360 (83.3)
≥7	42 (16.6)	30 (16.8)	72 (16.7)
Mode of delivery			
Vaginal**	18 (7.1)	89 (49.7)	107 (24.8)
Caesarean section	235 (92.9)	90 (50.3)	325 (75.2)
Fetal outcome			
Alive	140 (55.3)	78 (43.6)	218 (50.4)
Still birth	87 (34.4)	77 (43.0)	164 (38.0)
Early neonatal death*	26 (10.3)	24 (13.4)	50 (11.6)
Fetal sex			
Male	145 (57.3)	114 (63.7)	259 (60.0)
Female	108 (42.7)	65 (36.3)	173 (40.0)
Fetal weight (gm)			
1000–1499	21 (8.3)	24 (13.4)	45 (10.4)
1500–2499	72 (28.4)	45 (25.1)	117 (27.1)
2500–3999	154 (60.9)	104 (58.1)	258 (59.7)
4000+	6 (2.4)	6 (3.4)	12 (2.8)

*Within seven days of birth. **Including two vacuum and one forceps assisted delivery.

**Table 4:** Univariate analysis on perinatal mortality in relation to selected demographic and obstetric factors among women with antepartum haemorrhage (APH), 2006–2011, Hawassa University Hospital, Ethiopia.

Variable	Total APH* (*N* = 432)	Perinatal deaths (*N* = 214)	*P* value
Mothers' age in years			
<20	24	6 (25.0)	
20–34	321	166 (51.7)	0.04
35+	87	42 (48.3)	
Mothers' residence			
Urban	163	69 (42.3)	
Rural	269	145 (53.9)	0.02
Distance traveled in km			
<50	178	66 (37.1)	
≥50	254	148 (58.3)	<0.0001
Antenatal care			
Yes	283	124 (43.8)	
No	149	90 (60.4)	0.001
Parity			
0	87	31 (35.6)	
I–IV	204	104 (51.0)	0.04
V+	141	79 (56.0)	
Gestational age in weeks			
28–33	84	60 (71.4)	
34–36	127	52 (40.9)	<0.0001
37+	221	102 (46.2)	
Maternal anaemia on admission			
Severe	113	104 (92.0)	
Moderate	121	52 (43.0)	<0.0001
Mild	91	27 (30.0)	
No anaemia	107	31 (29.0)	
Fetal weight (gm)			
1000–1499	45	36 (80.0)	
1500–2499	117	66 (56.4)	<0.0001
2500–3999	258	112 (43.4)	
4000+	12	0	

*Placenta previa + placental abruption; APH: antepartum haemorrhage.

**Table 5:** Binary logistic regression on predictors of perinatal mortality in women with placenta previa and placental abruption, 2006–2011, Hawassa University Hospital, Ethiopia.

Variable	Crude		Adjusted**	
	*P* value	OR (95% CI)	*P* value	OR (95% CI)
Long distance traveled*	0.002	0.8 (0.63, 0.90)	0.31	1.0 (0.99, 1.01)
Lengthy delay before arrival*	0.005	1.01 (1.00, 1.02)	0.003	1.01 (1.005, 1.02)
Low gestational age*	<0.0001	1.1 (1.08, 1.22)	<0.0001	1.4 (1.16, 1.65)
No antenatal care	0.008	0.6 (0.39, 0.87)	0.07	0.34 (0.11, 1.09)
Low blood pressure*	<0.0001	1.02 (1.01, 1.03)	0.94	1.0 (0.98, 1.02)
Anaemia*	<0.0001	1.5 (1.41, 1.68)	<0.0001	1.9 (1.42, 2.59)
Male fetal sex	0.009	1.7 (1.14, 2.51)	<0.0001	11.8 (3.13, 44.25)
Vaginal delivery	<0.0001	1.4 (1.16, 1.58)	<0.0001	2.5 (1.55, 3.97)

*Continuous; all others are dichotomous.

**Adjusted for all variables in this table; other variables like maternal age, parity, and residence did not show significant association in the crude odds ratio.
